# Deducing the Temporal Order of Cofactor Function in Ligand-Regulated Gene Transcription: Theory and Experimental Verification

**DOI:** 10.1371/journal.pone.0030225

**Published:** 2012-01-17

**Authors:** Edward J. Dougherty, Chunhua Guo, S. Stoney Simons, Carson C. Chow

**Affiliations:** 1 Steroid Hormones Section, Clinical Endocrinology Branch, National Institute of Diabetes and Digestive and Kidney Diseases, National Institutes of Health, Bethesda, Maryland, United States of America; 2 Laboratory of Biological Modeling, National Institute of Diabetes and Digestive and Kidney Diseases, National Institutes of Health, Bethesda, Maryland, United States of America; Ecole Normale Supérieure de Lyon, France

## Abstract

Cofactors are intimately involved in steroid-regulated gene expression. Two critical questions are (1) the steps at which cofactors exert their biological activities and (2) the nature of that activity. Here we show that a new mathematical theory of steroid hormone action can be used to deduce the kinetic properties and reaction sequence position for the functioning of any two cofactors relative to a concentration limiting step (CLS) and to each other. The predictions of the theory, which can be applied using graphical methods similar to those of enzyme kinetics, are validated by obtaining internally consistent data for pair-wise analyses of three cofactors (TIF2, sSMRT, and NCoR) in U2OS cells. The analysis of TIF2 and sSMRT actions on GR-induction of an endogenous gene gave results identical to those with an exogenous reporter. Thus new tools to determine previously unobtainable information about the nature and position of cofactor action in any process displaying first-order Hill plot kinetics are now available.

## Introduction

Ligand-regulated gene induction is a productive experimental system for examining the mechanisms of gene expression. Steroid-regulated gene induction is an extensively studied paradigm with numerous well-defined events. The initially formed, intracellular receptor-steroid complex binds to specific enhancer-like DNA elements (called hormone response elements, or HREs) to eventually modify the rates of transcription of target genes. Other steps include recruitment of chromatin remodeling factors and cofactors that increase or decrease the rates of transcription [1]–[3].

One approach to defining steroid receptor actions at the molecular level has been to pair ChIP assays with genome-wide sequencing to identify all DNA binding sites of selected cofactors in the cellular genome [4]. Nonetheless, limitations to this approach remain. Not all factor binding sites are functionally active [5], [6]. The HREs may not regulate the closest gene [7], which is the default assignment. Methods to identify new cofactors are not generally available and the kinetic mechanism of most factors and cofactors is unknown. Because virtually all possible responses to steroid hormones have been observed with endogenous genes [8], no general mode of action exists. Finally, one can determine the temporal ordering of cofactor binding to DNA but no method exists to elucidate the temporal ordering of biological function. Cofactor binding to DNA is not equivalent to cofactor action. For example, paused RNA polymerase II is often present 50 bp downstream of the start of transcription but is not engaged in transcription [9]–[11]. Thus there is an unmet need for methods that can discern the precise nature (e.g., activator, non-competitive inhibitor) and temporal order of cofactor function.

Here we describe a method that establishes the functional mechanism and order of action of any active factor/cofactor relative to what we call a “concentration limiting step” (CLS). The method is derived from a recently developed theory of gene expression that is applicable to receptor-mediated transcriptional events that display a first-order Hill plot dose-response curve (FHDC) [12] for the gene product in experiments that reach equilibrium or steady state. The CLS is analogous to the rate-limiting step for a closed system and provides a reference point for the actions of all other cofactors. The method is based on analyzing graphs constructed from the maximal activity (A_max_) and potency (EC_50_) of the FHDC. Importantly, two cofactors can be assayed simultaneously and the graphical method determines the functional nature and order of both cofactors relative to each other and the CLS. This approach is amenable to screening cofactors because prior knowledge about their action is not required.

## Results

### Application of the theory to analyzing the actions of cofactors

The graphical method is derived from our steroid-mediated gene induction theory, which can generate a parametric model (i.e. formula) for the dose-response curve of the final protein product with respect to the steroid concentration in the presence of an arbitrary number of cofactors [12] (see Text S1 for the derivation). The theory considers a sequence of reaction steps, each with the form
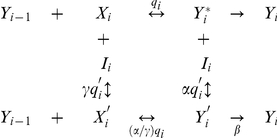
where *Y*_i_ is the reaction product of step *i*, *X*_i_ is an activating cofactor or activator, and *I*_i_ is an inhibiting cofactor or inhibitor. The labels on the reactions represent association constants for reversible reactions and reaction rates for nonreversible reactions. As in enzyme kinetics, we denote the case of 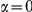 to be *competitive* inhibition, 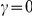 to be *uncompetitive* inhibition, 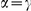 to be *noncompetitive* inhibition, and 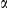 and 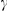 both nonzero to be *mixed* inhibition. The case of 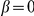 is called *linear* inhibition, and 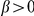 is called *partial* inhibition. In general, computing the dose-response curve for such a reaction sequence would be analytically intractable. However, imposing the experimentally observed constraint that the dose-response curve has a Hill-coefficient of one yields a closed-form expression for the dose-response curve in terms of the parameters of all the reactions.

The theory identifies a CLS, which is a step in the reaction sequence in which products of the reactions following it (post-CLS steps) are so small that the amounts of cofactor bound are negligible compared to their free concentrations [12]. A cofactor can act before, at, or after the CLS. It can be an activator or one of the four types of inhibitor and each inhibitor can be linear or partial. Although there are eight possible types of inhibitor (e.g. partial uncompetitive, linear noncompetitive, etc.), some combinations are not possible (e.g. a competitor cannot be partial). Inhibitors refer to their action in their particular reaction and not to the final product. For example, a partial inhibitor can give a higher response if it diverts the output to a higher yielding pathway. In fact, the action of a partial uncompetitive inhibitor is similar to an activator acting in a post-CLS step. For two cofactors, there are at most 285 possible cases accounting for where they act with respect to each other and the CLS, with each cofactor being either an activator or one of eight possible types of inhibitors. It should be noted that not all types of inhibitors are physically viable or can act in all locations. For example, among all types of inhibition, only competitive inhibition occurs after the CLS and partial competitive inhibition cannot exist. Each viable case is represented by an explicit parametric model that can be compared to the data (see Text S1).

We previously deduced that Ubc9 was an activator acting after the CLS by fitting parametric models directly to the data [12]. However, in that case there were only three models to test. For the more general case with multiple cofactors, directly fitting models to the data is unwieldy. However, the models have very different qualitative behaviors for different types of cofactors and their positions of action. In particular, the EC_50_ (steroid concentration required for half-maximal activity) and A_max_ (maximal activity) behave very differently with changing cofactor concentration. The graphical method exploits these differences to predict mechanism and position from the properties of *graphs* of functions of EC_50_ and A_max_ versus cofactor concentration. Hence, the cofactor mechanism and position of action is inferred from the qualitative behavior of how the dose-response curve changes and does not require making direct estimates of the parameter values.

### Graphical analysis of single cofactor actions

**Figure 1:**
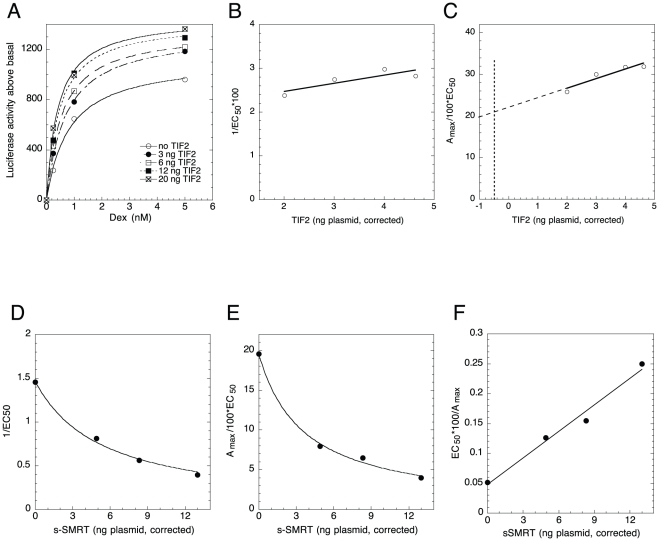
Analysis of actions of excess TIF2 or sSMRT. (A) Determination of A_max_ and EC_50_ by exact fit of gene induction response. Luciferase activity in transiently transfected U2OS cells (100 ng GREtkLUC reporter, 0.5 ng of GR, and the indicated amounts of TIF2 plasmid) is plotted against Dex concentration. The A_max_ and EC_50_ were determined from the best-fit curve to a first order Hill plot as described in Materials and Methods. Plots of 1/EC_50_ (B) and A_max_/EC_50_ (C) vs. TIF2, corrected for non-linear protein expression as described in the Text S1. The dashed line is the extrapolation of the linear best-fit of the data. The dotted line equals the position of the y-axis at “true zero” of no TIF2 in cells. Plots of 1/EC_50_ (D) and A_max_/EC_50_ (E) vs. sSMRT, corrected for non-linear protein expression. The A_max_ and EC_50_ for Dex induction of Luciferase activity in transiently transfected U2OS cells (100 ng GREtkLUC reporter, 0.5 ng of GR, 3 ng of TIF2 and the indicated amounts of sSMRT plasmid) was determined as in A. (F) A_max_/EC_50_ vs. corrected sSMRT approaches zero at infinite sSMRT. A linear plot of A_max_/EC_50_ vs. corrected sSMRT is diagnostic of an asymptote value equal to zero at infinite sSMRT. A positive asymptote value would give a non-linear, downward curving plot that can be linearized by subtracting the estimated asymptote from each of the values for A_max_/EC_50_ in panel E and then plotting the reciprocal (1/[(A_max_/EC_50_) – asymptote]). The combined results from this one representative experiment were seen in three other independent experiments.

We applied our method to two well-known cofactors for glucocorticoid receptor (GR, also called NR3C1) transactivation: the coactivator TIF2 and the corepressor SMRT [13], [14]. To obtain easily quantifiable data for graphical analysis, the induction of a synthetic reporter gene (GREtkLUC) by GRs is followed in transiently transfected cells with different cofactor concentrations and triplicate sub-saturating concentrations of the glucocorticoid dexamethasone (Dex). The A_max_ and EC_50_ are abstracted from fits of the data points to a first order Hill function (**Fig. 1A**). Graphs of 1/EC_50_ vs. concentration and A_max_/EC_50_ are then plotted.

Figure 1B shows the plot of 1/EC_50_ vs. transfected TIF2 plasmid (after correction for frequent non-linear expression of plasmid-encoded protein [see Text S1 and **Fig. S1**]) for an experiment with four TIF2 concentrations. The curve is seen to be increasing and according to the theory, the only possible parametric models are a straight line with a positive slope or a nonlinear rational function. The data are seen to be consistent with being linear. Although it is true that a nonlinear function is possible it is far less parsimonious. We thus first assume that the curves are linear and check for logical consistency with the other predictions (see Text S1 for full algorithm to determine shape of the curves). If linearity fails to give a logically consistent prediction then we can test the hypothesis that the curves are nonlinear. According to **Table 1**, linear with positive slope is characteristic of TIF2 acting in one of three manners. (Note: all below graphs are for corrected non-linear protein expression.) The interpretation of the linear plot of A_max_/EC_50_ vs. TIF2 (**Fig. 1C**) depends upon the y-axis intercept when the total TIF2 (endogenous plus added) equals zero. The relative amount of endogenous TIF2 was determined by Western blotting to be equivalent to 0.97 ng of transfected TIF2 plasmid (not shown). Experiments with fluorescent-tagged proteins indicated that ∼50% of the cells are transfected. Thus, endogenous TIF2 in transfected cells equals about 0.49 ng TIF2 plasmid. The average x-axis intercept from 4 experiments like Fig. 1C is −5.6±3.6 (S.D.) ng of TIF2 plasmid. Therefore the A_max_/EC_50_ plots intersect the x-axis at less than “true zero” (i.e., less than no endogenous TIF2). Consequently the y-axis intercept is >0. From Table 1A, we now conclude that TIF2 is an activator after the CLS or the mathematically indistinguishable case of a partial uncompetitive inhibitor before the CLS.

**Table 1:** Algorithms for single factor plots for factor F.

Plot parameters	Plot properties	Mechanistic conclusions
1/EC_50_ vs. F	linear with zero slope (i.e. does not change with F)	1) F = A at CLS or 2) F = PN before CLS
	linear with positive slope	1) F = A not at CLS; or 2) F = U before or at CLS, or 3) F = PU before CLS
	nonlinear decreasing curve (concave-up)	1) F = C, 2) F = M before or at CLS
	nonlinear increasing curve (concave-down)	1) F = LM or PM, before CLS; or 2) F = M at CLS
A_max_/EC_50_ vs. F	linear; y-axis intercept = 0	1) F = A before or at CLS
	linear; y-axis intercept >0	1) F = A after CLS; or 2) F = PU before or at CLS
	nonlinear decreasing curve that approaches zero for large F	F = C before or at CLS
	nonlinear decreasing curve that approaches positive value for large F	1) F = PM or PN, before or at CLS; or 2) F = C after CLS
	nonlinear increasing curve	1) F = PM or PN, before or at CLS
EC_50_/A_max_ vs. F	linear with positive slope	1) F = C before or at CLS

The originally described form of SMRT, sSMRT [15], lacks about 1000 amino-terminal residues [16]. Both sSMRT and full length SMRTs are corepressors of steroid receptor action. Graphical analysis of sSMRT as above with a constant, low amount of exogenous TIF2 (to increase the starting signal), yields a decreasing curve for 1/EC_50_ vs. sSMRT (**Fig. 1D**). The only allowable form of this curve by the theory is a decreasing nonlinear first-order decay plot. The plot of A_max_/EC_50_ is also first-order decay (**Fig. 1E**). The important characteristic of decaying curves is whether they decay to zero or a positive value asymptotically. This can be determined graphically by plotting the reciprocal function (EC_50_/A_max_) and seeing if it is linear, which is confirmed in **Fig. 1F**. The only interpretation in Table 1 compatible with Figs. 1D–F is that sSMRT is a competitive inhibitor before or at the CLS.

### Graphical analysis of dual cofactor actions (TIF2 vs. sSMRT)

Our method can simultaneously analyze two cofactors and determine the site of each cofactor's action relative to the CLS, and usually relative to each other (see **Table S1**). Four plots (1/EC_50_ and A_max_/EC_50_ for each cofactor), plus information on plasmid expression efficiency, are needed for maximal information, often with quantitation of endogenous cofactor. More than one classification of each plot may appear possible. However, by eliminating incompatible mechanistic consequences, one almost always reaches a single, internally logically consistent mechanistic description. Favorable conditions are with two cofactors of opposite activities, thereby yielding the greatest changes. As TIF2 and sSMRT fit these criteria, we analyzed their actions using four concentrations of each cofactor in all combinations, plus a control of no added cofactors.

**Figure 2:**
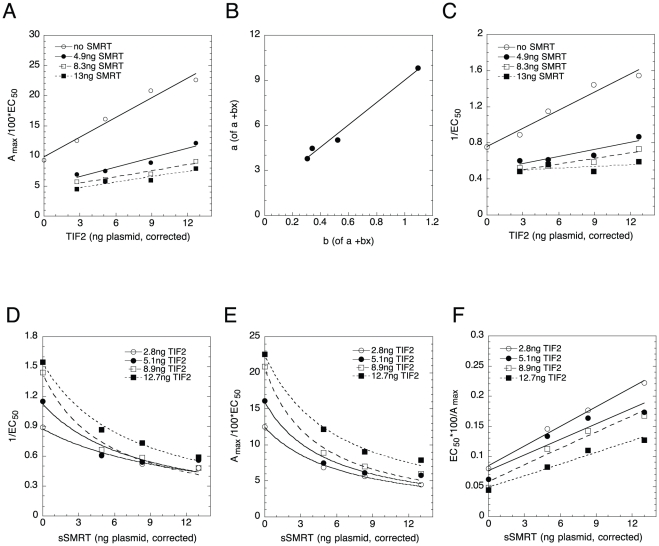
Analysis of combined actions of TIF2 and sSMRT. (A) Graph of A_max_/EC_50_ vs. TIF2. The results of one representative experiment (n = 4), conducted as in Fig. 1, are shown for the indicated amounts of transfected TIF2 plasmid, corrected for non-linear protein expression. (B). Determination of intersection coordinates. The coefficients “a” and “b” of the linear plots in A, described by y = a+bx, are graphed. The negative value of the slope, and y-axis intercept, in this graph give the x- and y-axis the values respectively for intersection point of the lines in A, as described in the text and Text S1. Data for one representative graph each (n = 4) of 1/EC_50_ vs. TIF2 (C) or sSMRT (D), of A_max_/EC_50_ vs. sSMRT (E) and of EC_50_/A_max_ vs. sSMRT (F) were acquired as in A and plotted against the indicated amounts of transfected plasmids, each of which have been corrected for non-linear protein expression.

Figure 2A shows that plots of A_max_/EC_50_ vs. TIF2 plasmid for increasing sSMRT consists of lines with progressively lower positive slopes. Western blots indicated that endogenous TIF2 in these experiments equaled 4.1 ng of TIF2 plasmid (data not shown). Entries 23,25, 28, and 29 of Table S1 are consistent with these graphs. Deciding between these entries for **Fig. 2A** depends upon the x- and y-axis coordinates of the intersection point of the lines. The x-axis value was determined, from what we call an “a vs. b plot” (**Fig. 2B**; see Methods), to be more negative (Ave. = −8.6±1.4, S.D., n = 4) than the endogenous TIF2 (−4.1), while the y-axis value was 2.3±2.2. This restricts the possible graphical interpretations to entries 25 and 29. Coupled with the unique assignment of entry 7 to 1/EC_50_ vs. TIF2 plots (**Fig. 2C**; x-axis intercept = −8.9±3.0)), the possible behavior of TIF2 and sSMRT reduces to: TIF2 is an activator acting after the CLS, sSMRT is a competitive inhibitor acting at the CLS and before TIF2. The other plots of 1/EC_50_ vs. sSMRT (**Fig. 2D**), A_max_/EC_50_ vs. sSMRT (**Fig. 2E**), and EC_50_/A_max_ vs. sSMRT (**Fig. 2F**) can each be described by entries 18, 32, and 37 respectively with the same unique mechanistic interpretation as above.

### Graphical analysis of TIF2 and NCoR competition

**Figure 3:**
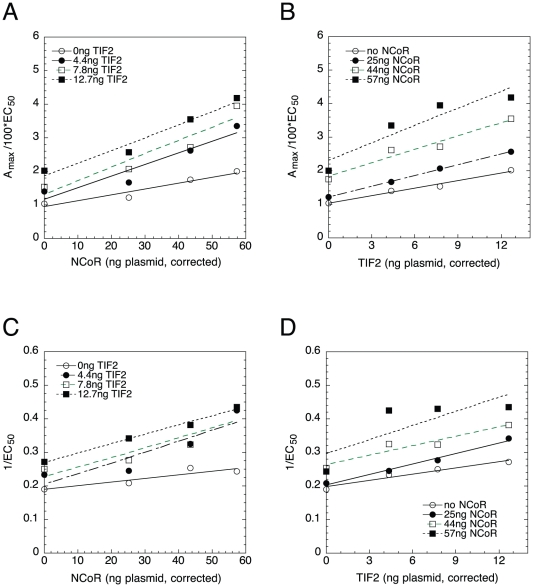
Analysis of combined actions of TIF2 and NCoR. Graphs of A_max_/EC_50_ vs. NCoR (A) and TIF2 (B) and of 1/EC_50_ vs. NCoR (C) and TIF2 (D). The results of one representative experiment (n = 6), conducted as in Fig. 1, are shown for the indicated amounts of transfected TIF2 and NCoR plasmids, corrected for non-linear protein expression.

NCoR is another well-documented corepressor that is thought to act like sSMRT [13], [14]. We investigated the actions of NCoR competing with TIF2 under the conditions of Fig. 2. Western blots were used to correct for non-linear expression and to determine relative endogenous TIF2 (4.1 ng TIF2 plasmid) and NCoR (8.3 ng NCoR plasmid) levels. Given these values, only entries 26 or 27 can describe the graph of A_max_/EC_50_ vs. NCoR (**Fig. 3A**), with calculated x- and y-intersection points of −30.0±26.7 and 1.2±1.0 (S.D., n = 6) respectively. Thus NCoR is an activator acting after the CLS and TIF2 is an activator acting after the CLS. Each of the other graphs has more than one potential classification due to insufficient precision in the graphical intersection points: A_max_/EC_50_ vs. TIF2 (**Fig. 3B**; entries 24 and 27), 1/EC_50_ vs. NCoR (**Fig. 3C**; 6, 7, and 11), and 1/EC_50_ vs. TIF2 (**Fig. 3D**; entries 5–7 and 11). However, most graphical options are incompatible with both factors being activators after the CLS and can be eliminated to yield: NCoR is an activator acting after the CLS, TIF2 is an activator after the CLS, and TIF2 and NCoR do not act at the same step. While these data are not yet able to determine whether TIF2 is an activator after or before NCoR, the conclusion that TIF2 is an activator after the CLS is identical to the above results with TIF2 vs. sSMRT.

### Graphical analysis of sSMRT and NCoR competition

**Figure 4:**
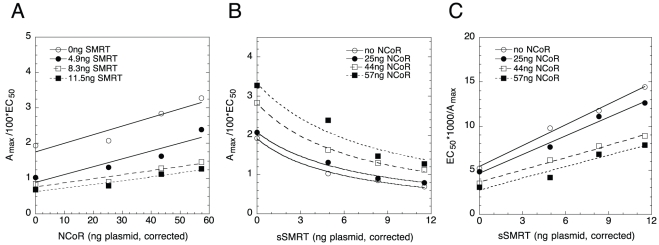
Analysis of combined actions of NCoR and sSMRT. Graphs of (A) A_max_/EC_50_ vs. NCoR, (B) A_max_/EC_50_ vs. sSMRT, and (C) EC_50_/A_max_ vs. sSMRT. The results of one representative experiment of a total of three independent experiments, conducted as in Fig. 1, are shown for the indicated amounts of exogenous NCoR and sSMRT plasmids, after correction for non-linear protein expression.

To confirm the above different actions of sSMRT and NCoR, we directly competed the activities of both cofactors under conditions of Fig. 2. Here the key graphs again involve A_max_/EC_50_. For NCoR (**Fig. 4A**), the calculated x-axis intersection of −74±31 is clearly less than the −8.3 of endogenous NCoR while the y-axis intersection is −0.04±0.4 (S.D., n = 3). These properties are uniquely defined by entry 25. Deciding whether A_max_/EC_50_ vs. sSMRT (**Fig. 4B**) is described by entry 32 or 33 is resolved by the linear plots of EC_50_/A_max_ (**Fig. 4C**). This characteristic of entry 37 is obtained only when nonlinear decreasing A_max_/EC_50_ plots approach zero with infinite F2. At this point, we can conclude that: NCoR is an activator after the CLS, sSMRT is a competitive inhibitor before or at the CLS, and NCoR acts after sSMRT. The 1/EC_50_ graphs with either factor are consistent with several interpretations (entries 8–10 and 12 for NCoR and 18 and 19 for sSMRT; **Fig. S2**), one of which is, for each factor, the same as that derived from the other graphs. It is not possible with these data, though, to determine whether sSMRT acts before or at the CLS.

### Graphical analysis of TIF2 and sSMRT competition for induction of an endogenous gene

**Figure 5:**
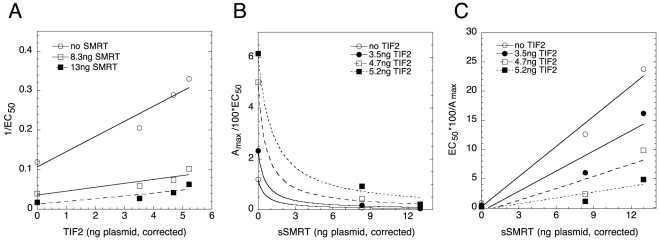
Analysis of combined actions of TIF2 and sSMRT for induction of the endogenous gene, IGFBP1. Plots of (A) 1/EC_50_ vs. TIF2, (B) A_max_/100×EC_50_ vs. sSMRT, and (C) EC_50_/A_max_ vs. sSMRT. The results of one representative experiment of a total of four independent experiments, conducted similarly as in Fig. 1, are shown for the indicated amounts of transfected NCoR and sSMRT plasmids, after correction for non-linear protein expression. The differences are that IGFBP1 mRNA (instead of luciferase) was determined by qRT-PCR and fold-induction is used in place of A_max_ (see text).

The relevance of the above competition of TIF2 and sSMRT, and the applicability of the graphical analysis to normal cellular biology, was next examined in the context of the GR-inducible IGFBP1 gene in U2OS cells [17]–[19]. qRT-PCR quantitation of IGFBP1 mRNA induction employed SyberGreen, which gives relative total activities. Therefore, the A_max_ in these experiments is actually the closely related fold-induction above basal level. 1/EC_50_ vs. TIF2 graphs (**Fig. 5A**) are exclusively described by entry 9 because the “a vs. b plots” give an x-axis intersection point (−3.5±0.6, S.D., n = 4) that is much more negative than the endogenous TIF2 in these experiments of −0.49. The non-linear A_max_/EC_50_ (**Fig. 5B**), with the linear EC_50_/A_max_ (**Fig. 5C**), graphs vs. sSMRT implicate entries 32 and 37 respectively, which define the factors as: TIF2 is an activator after the CLS, sSMRT is a competitive inhibitor at the CLS, and TIF2 acts after sSMRT. The predictions from the other graphs (A_max_/EC_50_ vs. TIF2 and 1/EC_50_ vs sSMRT; **Fig. S3**) are entirely consistent with this interpretation. Gratifyingly, this conclusion is the same as seen above for these factors with an exogenous reporter gene.

## Discussion

This report describes how a new mathematical theory of steroid-induced gene transcription [12] gives previously unobtainable mechanistic information about GR- (NR3C1-) regulated gene induction. This information could be obtained from direct curve fitting of predictions from our theory but it would be extremely computationally intensive. Instead, we carefully examined the underlying equations of the theory. This analysis unexpectedly revealed that plots used in enzyme kinetics can be adapted to analyze the role of cofactors in GR-mediated gene transactivation. These graphical methods give information regarding the specific cofactor function and also the location in the reaction scheme where the cofactor is acting relative to a CLS. A major advantage of these graphical methods is that they are readily employed without the needs of elaborate and extensive mathematical calculations. Our method works with a single added cofactor but is more informative when two cofactors are examined together, in which case the nature and location of action of both cofactors can be determined simultaneously. Even if a precise mechanistic interpretation is not possible, differences in the graphical analyses can reveal mechanistic non-equivalence of two cofactors that otherwise may be thought to share a common mode of action.

Several approaches were taken to validate our graphical analysis using GR-regulated gene induction as the model system. The p160 family member, TIF2, is a well-documented coactivator of steroid receptors [13], [20], [21]. In three different systems, TIF2 was always a activator acting after the CLS. Likewise, the corepressor sSMRT [13], [20], [21] was always found to be a competitive inhibitor, acting at the CLS in two systems and at or before the CLS in the third. To our knowledge, this is the first identification regarding either the kinetics of mechanism, or placement of action, of TIF2 or sSMRT.

A major test of our graphical methods came upon examining the actions of three cofactors (TIF2, sSMRT, and NCoR) in all possible pair-wise combinations. The mechanism and site of action of each cofactor was the same in competition assays with both of the other two cofactors. This internal logical consistency of cofactor mechanism and site of action strongly validates our new method. It also suggests that there is just one CLS, which does not change with assay conditions. This is important in ascertaining whether the CLS is invariant and a common marker step when comparing the actions of different cofactors.

Our extended model and associated graphical analysis also works with endogenous genes. The fact that the mechanisms of TIF2 and sSMRT in IGFBP1 induction involve identical sites of action as seen with the transiently transfected GREtkLUC reporter is further support for both the utility of our method and for the invariance of the CLS. Also, it has long been assumed that most steps of GR-regulated gene induction are the same for exogenous and endogenous genes. We recently demonstrated that the modulatory activity of TIF2 is very similar with both types of reporters [22]. The present data suggest that the mode and site of TIF2 action may also be the same with both classes of reporters.

**Figure 6:**
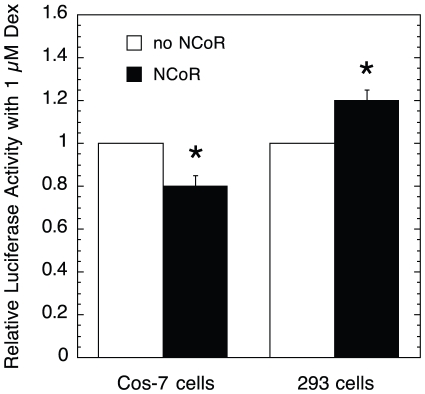
Cell-selective activity of NCoR. Cos-7 or 293 cells were transiently transfected with the same amount of plasmids for glucocorticoid receptor (0.5 ng), NCoR (90 ng) or equimolar amount of control plasmid (51.6 ng of human serum albumin in the pCMX vector), and GREtkLUC reporter and induced with EtOH ±1 µM Dex as described in Materials and Methods. The total Luciferase activity with 1 µM Dex with each treatment was expressed relative to that with no added NCoR (± S.D., n = 3). P values: *≤0.021.

The different manners of NCoR and sSMRT action was unexpected. NCoR and sSMRT are generally thought to act as corepressors by binding to nearly the same site as coactivators, thereby excluding coactivator binding [23], [24]. Not only does our graphical analysis indicate different mechanisms of action at different steps for NCoR and sSMRT but also NCoR, being kinetically defined as an activator, could be called a coactivator because it increases the A_max_ in U2OS cells. However, this property of NCoR is cell-dependent, with NCoR decreasing the A_max_ from GREtkLUC in Cos-7 cells and increasing the A_max_ in 293 cells (**Fig. 6**). Such cell-selective differences are not unique and have been seen not only for sSMRT with estrogen receptors in HeLa cells [25], and GR in CV-1 vs. 1407.2 cells and progesterone receptors in 1470.2 cells [26], but also with ZAC1b [27] and CIA with estrogen receptors [28], and with SRAP activation of androgen receptors and repression of VP16 transactivation [29]. In a recent report, 19 of 25 cofactors examined have dual activity and can increase and decrease the activity of agonist steroids with the androgen receptor in a gene-dependent manner [30]. It will be interesting to use our graphical analysis to determine the precise kinetic properties of the context-dependent activities of these versatile cofactors.

Our theory and methods are not without limitations. Any process not displaying FHDC kinetics of induction or repression cannot be analyzed. Conversely, our approach is applicable to any process yielding FHDC kinetics such as hormones in general [31], G-protein mediated responses [32], [33], the developmental-specific genes of Drosophila embryogenesis [34], and glucocorticoid-induced apoptosis of thymocytes [35]. While we have yet to discover a straightforward method to determine the graphical consequences of every mechanistic combination, the most common mechanisms listed in Table S1 cover over 74% of the possible cases. Thus, some graphical patterns may emerge that have not yet been linked to a specific mechanism. Nonetheless, the current range of analyzable mechanisms offers a powerful method for determining two aspects of steroid hormone action for which no alternative method presently exists: the kinetic actions of cofactors and the relative ordering of cofactor activity in the overall pathway. With this last capability, it is now theoretically possible to construct an ordered sequence based on the biological function of cofactors, much as in the ordering of pathways by epistasis analysis, even if the biochemical properties of the cofactors are not known. As a start, the present experiments in U2OS cells reveal that TIF2 and NCoR act at different steps after the CLS and after sSMRT, which acts at the CLS. This type of information should be useful in identifying downstream steps for therapeutic intervention, thereby reducing the number of side-effects that accompany the inhibition of upstream steps in steroid hormone action.

## Materials and Methods

Unless otherwise indicated, all cell growth was at 37°C and all other operations were performed at r.t.

### Chemicals

Dexamethasone (Dex) was purchased from Sigma (St. Louis, MO). Dex-21-mesylate (DM) was from Steraloids (Newport, RI). Anti-TIF2 mouse monoclonal antibody (No. 610984; BD Biosciences, San Jose, CA), anti-β actin monoclonal antibody (Sigma, St. Louis, MO), and goat anti-mouse horseradish peroxidase (Santa Cruz Biotechnology, Inc., Santa Cruz, CA) are commercially available. Rabbit anti-NCoR antibody was a gift from Goeffrey Rosenfeld (UC San Diego, CA).

### Plasmids

The GREtkLUC reporter is a synthetic plasmid with a tandem repeat of the second glucocorticoid response element (GRE) of the rat tyrosine aminotransferase gene fused upstream of the thymidine kinase (tk) promoter driving the firefly luciferase (LUC) gene [36]. Renilla TS is a gift from Nasreldin M. Ibrahim, Otto Fröhlich, and S. Russ Price (Emory University School of Medicine), pSG5/TIF2 is from Heinrich Gronemeyer (IGBMC, Strasbourg, France), pCMX/sSMRT is from Ronald Evans (Salk Institute, La Jolla, CA), NCoR/Flag is from Geoff Rosenfeld (University of California-San Diego, San Diego, CA), and rat GR/pSG5, pSG5/human serum albumin, pCMX/human serum albumin, and pBSK have been previously described [37]–[40]. The Renilla null luciferase reporter is from Promega (Madison, WI).

### Cells and Growth Conditions

U2OS human osteosarcoma cells (from ATTC, #HTB-96) were grown in high glucose DMEM (Invitrogen, Carlsbad, CA) with 10% fetal bovine serum as previously described for U2OS cells with stably transfected GR (17). Cells were split at 3- to 4-day intervals and used in experiments at approximately 60–90% confluence. For steroid treatments, Dex and DM solutions were prepared in 100% ethanol and diluted ≥1∶100 into growth medium. Growth medium for the cells was then replaced with ethanol- or steroid-medium for the indicated incubation time.

### Transient Transfection and Reporter Analysis

U2OS cells (20,000 cells per well) were seeded 24 h before transfection in 24-well plates. GREtkLUC reporter plasmid (100 ng/well) and other plasmids (total DNA adjusted to 300 ng/well with pBluescriptII SK+ [Stratagene, Santa Clara, CA]) were transiently transfected with Lipofectamine 2000 (Invitrogen, Carlsbad, CA) according to the manufacturer's instructions. Cells were treated 24 h post-transfection with steroids for 16–20 h and assayed for luciferase activity using a dual-luciferase reporter assay (Promega, Madison, WI) on a Centro XS^3^ LB 960 luminometer (Berthold Technologies, Oak Ridge, TN). Luciferase activity was normalized to Renilla TS as an internal control.

### Quantitative Real-Time PCR (qRT-PCR)

U2OS cells (150,000–200,000 cells per well) were seeded 24 h before transfection in 6-well plates. Plasmids (total DNA = 1500 ng/well) were transiently transfected with Lipofectamine 2000 (Invitrogen) according to the manufacturer's instructions in the same ratios as used for 24-well dishes. Cells were treated 24 h post-transfection with various concentrations of Dex and DM for 20 h. Total RNA was extracted using TriZol reagent (Invitrogen) and cDNA was synthesized using SuperScript III, First-Strand Synthesis Kit (Invitrogen) per manufacturer's recommendations. The relative expression level of the corresponding cDNA was quantified using SyberGreen in an ABI 7900HT real-time PCR system. The quantification was normalized against β-Actin using the 2^−ΔΔCT^ method. For all qRT-PCR reactions, primer efficiencies were 100% (±10%), appropriate no-RT and template free controls were used, and primer melting curves were assessed to ensure specificity of the PCR products.

### Western Blotting

U2OS cells were transiently transfected with TIF2 expression plasmid under conditions identical to those above. After 48 h, cells were lysed using Cytobuster Protein Extraction Reagent (EMD Chemicals, Gibbstown, NJ) and equal amounts of lysate run on 4–12% Bis-Tris NuPAGE gels (Invitrogen) per manufacturer's instructions. Western blots were prepared, probed with mouse anti-TIF2 or mouse anti-β actin antibody followed by goat anti-mouse horseradish peroxidase. Bands were visualized with enhanced chemiluminescence detection reagents as described by the manufacturer (GE Healthcare, Piscataway, NJ). The relative concentration of target proteins was determined by densitometric analysis of the exposed film.

### Data analysis

Statistical significance for Luciferase activity is assessed by the two-tailed Student's t test using InStat 2.03 (GraphPad Software, San Diego, CA). Each average of triplicates is treated as one value of the *n* experiments. When the difference between the SDs of two populations is significantly different, the t test is invalid so the Mann-Whitney or Alternate Welch t-test is used. A nonparametric test is used if the distribution of SD values is non-Gaussian.

The maximum induced activity (A_max_) was obtained with saturating concentrations of agonist steroid, which was the lower of ≥100-fold higher than the EC_50_ or 10 µM. One dose-response curve yields one value of EC_50_ via a curve-fitting program (KaleidaGraph; Synergy Software, Reading, PA) following a first order Hill plot for increase or decay (R^2^ almost always ≥0.95). Alternatively, we can determine A_max_ and EC_50_ directly by fitting the curve
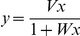
where V = A_max_/EC_50_ and W = 1/EC_50_.

### Graphical analysis

After obtaining A_max_/EC_50_ and 1/EC_50_ from the dose response curve, graphs of A_max_/EC_50_ vs. C and 1/EC_50_ vs. C, where C is the concentration of a cofactor, were constructed. In experiments where two cofactors were assayed, graphs of A_max_/EC_50_ vs. C and 1/EC_50_ vs. C for each cofactor for varying concentrations of the other cofactor would be made. Thus, if an experiment used *n* concentrations for each cofactor, then there would be a total of four to six graphs, each with *n* separate curves, that are analyzed as described in the Text S1 and Fig. S4. The shape of the curves and how they change with the other cofactor are then compared to Table 1 for a single cofactor and Table S1 for two cofactors to determine the mechanism and order of action. Some entries in the table require knowledge of the endogenous concentration in the cell because the value at zero total concentration is required. This can be obtained from Western blots.

Many of the entries in Table S1 require an estimate of the intersection point of a set of linear regression fits to the graphs. We used the following method to make this estimate. Consider a family of lines 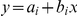 obtained from the linear regression fits for each graph labeled by *i*. The intersection point occurs if there exists a point (*x*,*y*) that simultaneously satisfies this system of equations. In general, because of noise and measurement error, the lines will not intersect exactly. Solving the system simultaneously for the intersection point is not viable because the system will generally be singular. However, rearranging the linear system yields 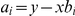 and a linear regression on the graph of *a* vs *b*, will give the least squares maximum likelihood estimate for the intersection points *x* and *y*. The *a*-intercept of the linear regression fit corresponds to the *y* value, and the negative slope corresponds to the *x* value, of the intersection point. The errors in the *a* vs *b* linear regression give an indication of the likelihood that the lines do in fact intersect.

## Supporting Information

Figure S1 **Linearization of transfected plasmid concentrations.** (A) Plot of normalized OD of Western blots (above endogenous TIF2) for different amounts of transfected TIF2 plasmid (but same amount of cell lysate protein) vs. OD of maximum amount of transfected TIF2 (20 ng). (B) Plot of normalized ODs of transfected TIF2 (above endogenous TIF2) vs. “linearized plasmid”. Amounts (ng) of linearized TIF2 plasmid were calculated as described in the Supplementary Material and then plotted against the normalized OD values of Fig. S1A. Error bars = S.D. (n = 2, each in duplicate).
(EPS)

Figure S2 **Plots of 1/EC_50_ vs. cofactor for experiment of** **Fig. 4** **.** (A) 1/EC_50_ vs. sSMRT plasmid (corrected for non-linear expression) with different amounts of competing NCoR plasmid. (B) 1/EC_50_ vs. NCoR plasmid (corrected for non-linear expression) with different amounts of competing sSMRT plasmid.
(EPS)

Figure S3 **Plots of EC_50_ and A_max_ vs. cofactor for experiment of** **Fig. 5** **with endogenous IGFBP1 gene.** (A) 1/EC_50_ vs. sSMRT plasmid (corrected for non-linear expression) with different amounts of competing TIF2 plasmid. (B) A_max_/EC_50_ vs. TIF2 plasmid (corrected for non-linear expression) with different amounts of competing sSMRT plasmid.
(EPS)

Figure S4 **Cartoon depicting process of restricting mechanistic scenarios of the six graphs used in analyzing the competition assays.** Each circle represents one type of graph (labeled on perimeter) and is composed of different mechanistic scenarios (e.g., Scenario A for 1/EC_50_ vs. F1) for that graph (see Table 1). The area of overlap for all graphs (depicted by the filled space in the cartoon) represents the common, uniquely identified mechanism for that specific competition assay. This 4- to 6-fold requirement of “overlap” is a stringent test for determining the mechanistic explanation of any given competition assay.
(EPS)

Table S1 Algorithms for two factor plots for factors F1 and F2.
(DOCX)

Text S1 Derivation of the graphical method for analyzing the competitive action of factors and Correction for non-linear protein expression from transfected plasmids.
(DOCX)
