# Supplementary material for: Deducing the Temporal Order of Cofactor Function in Ligand-Regulated Gene Transcription: Theory and Experimental Verification
Source: PLoS One. 2012 Jan 17;7(1):e30225. doi: 10.1371/journal.pone.0030225 (PMC3260260; doi:10.1371/journal.pone.0030225)
Supplement: Table S1 — Algorithms for two factor plots for factors F1 and F2. (DOCX) [file pone.0030225.s005.docx]

Table 2: Algorithms for two factor plots for factors F1 and F2

| Entry No. | Plot parameters | Plot properties | Mechanistic conclusions |
| --- | --- | --- | --- |
| 1 | 1/EC_50_ vs. F1 for different values of F2 | linear with zero slope;  curves do not change with F2 | 1) F1 = PN before CLS and F2 = A at CLS |
| 2 |  | linear with zero slope;  y-axis intercept increases with F2 | 1) F1 = A at CLS and ( a) F2 = A not at CLS or b) F2 = U or M at CLS or c) F2 = U or M or PN before CLS); or 2) F1 = PN before CLS and ( a) F2 = A not at CLS, or b) F2 = U or M at CLS, or c) F2 = U or M or PN, before CLS) |
| 3 |  | linear with zero slope;  y-axis intercept decreases with F2 | 1) F1 = A at CLS and  a) F2 = C or M, before or at CLS  b) F2= C after CLS |
| 4 |  | linear; slope increases;  curves do not change with F2 | 1) F1 = A not at CLS and F2 = A at CLS; or  2) F1 = U before or at CLS and F2 = A at CLS; or 3) F1 = PU before CLS and F2 = A at CLS |
| 5 |  | linear; slope increases with F2; lines intersect at F1 = 0 | 1) F1 = A before CLS and ( a) F2 = A after F1 and not at CLS, or b) F2 = U or M, after F1 and at CLS, or c) F2 = PU or PM, after F1 and before CLS) |
| 6 |  | linear; slope increases with F2; lines intersect at F1 < 0 | 1) F1 = A before CLS and F2 = A before F1; or  2) F1 = PU before CLS and F2 = A not at CLS; or 3) F2 = U after F1 and before or at CLS; or  3) F1 = U at CLS and F2 = A before F1; or 4) F1 = A after CLS and F2 = A before CLS |
| 7 |  | linear; slope increases with F2; lines do not intersect at one point | 1) F1 = A before CLS and F2 = PU before or at F1; or 2) F1 = PU before CLS and F2 = PU before CLS; or 3) F1 = U at CLS and F2 = PU before F1 |
| 8 |  | linear; slope decreases with F2; lines intersect at F1 = 0; | 1) F1 = A before CLS and ( a) F2 = C after F1,  or b) F2 = M after F1 and before or at CLS); or 2) F1 = A after CLS and F2 = C at F1 |
| 9 |  | linear; slope decreases with F2; lines intersect at F1 < 0; | 1) F1 = PU before CLS and ( a) F2 = C after F1, or b) F2 = M after F1 and before or at CLS); or 2) F1 = A after CLS and F2 = C at CLS or C at F1 |
| 10 |  | linear; slope decreases with F2; lines do not intersect at one point | 1) F1 = A before CLS and F2 = C or M, before or at F1; or 2) F1 = PU before CLS and F2 = C or M, before F1; or 3) F1 = U at CLS and F2 = C or M, before F1; or 4) F1 = A after CLS and F2 = C or M, before CLS |
| 11 |  | linear; y-axis intercept increases with F2; lines never intersect (slopes of lines are the same) | 1) F1 = A after CLS and ( a) F2 = U at CLS, or b) F2= A after CLS); or 2) F1 = LU before CLS and ( a) F2 = A before CLS and after F1, or b) F2 = U or M, before or at CLS after F1); or 3) F1 = PU before CLS and F2 = LU before F1; or 4) F1 = U at CLS and F2= LU before F1 |
| 12 |  | linear; y-axis intercept decreases with F2; lines never intersect (slopes of lines are the same) | 1) F1= A after CLS and F2 = C after CLS not at F1; or 2) F1 = LU before CLS and F2 = C or M, before or at CLS and after F1 |
| 13 |  | nonlinear increasing, curves do not change with F2 | 1) F1 = LM or PM, before CLS and F2 = A at CLS; or 2) F1 = M at CLS and F2 = A at CLS |
| 14 |  | nonlinear increasing curve; curve position increases with F2 (shape not preserved) | 1) F1 = LM before CLS and ( a) F2 = A before F1, or b) F2 = PU or PM, before F1); or 2) F1 = M at CLS and ( a) F2 = A before F1, or b) F2 = PU or PM before F1); or 3) F1 = PM before CLS and ( a) F2 = A after F1, or b) F2 = U or M, after F1 at or before CLS) |
| 15 |  | Nonlinear increasing curve; curve position increases with F2 while preserving shape | 1) F1 = M before or at CLS and F2 = LU before F1 |
| 16 |  | nonlinear increasing curve; curve position decreases with F2 | 1) F1 = LM before CLS and F2 = C or M, before F1; or 2) F1 = PM before CLS and ( a) F2 = C or M, before CLS, or b) F2 = C at or after CLS); or 3) F1 = M at CLS and F2 = C before or after CLS |
| 17 |  | nonlinear decreasing, curves do not change with F2 | 1) F1=C or M, before or at CLS and F2 = A at CLS; or 2) F1=C after CLS and F2 = A at CLS |
| 18 |  | nonlinear decreasing curve; curve position increases with F2 | 1) F1 = C after CLS and ( a) F2 = A at F1, or b) F2 = A or PM or PU, before CLS); or 2) F1 = C at CLS and ( a) F2 = A before or after CLS, or b) F2 = PU or PM, before CLS); or 3) F1 = C or M, before CLS and ( a) F2 = A before or after CLS, or b) F2 = U after F1 and before or at CLS, or c) F2 = PU before F1) |
| 19 |  | nonlinear decreasing curve; curve position increases with F2 while preserving shape | 1) F1 = C after CLS and ( a) F2 = A after CLS and not at F1, or b) F2 = U at CLS, or c) F2 = LU before CLS); or 2) F1 = C or M, before or at CLS and F2 = LU before F1 |
| 20 |  | nonlinear decreasing curve; curve position decreases with F2 | 1) F1 = C after CLS and F2 = C or M, before or at CLS; or 2) F1 = C at CLS and ( a) F2 = C after CLS, or b) F2 = C or M, before CLS); or 3) F1 = C or M, before CLS and ( a) F2 = C or M, before or at CLS, or b) F2 = C after CLS) |
| 21 |  | nonlinear decreasing curve; curve position decreases with F2 while preserving shape | 1) F1 = C after CLS and F2 = C after CLS |
|  |  |  |  |
| 22 | A_max_/EC_50_ vs. F1 for different values of F2 | linear; slope increases with F2; lines intersect at y=0, y-axis intercept = 0 | 1) F1 = A at or before CLS and ( a) F2 = A, or b) F2 = PU or PM, before or at CLS) |
| 23 |  | linear; slope decreases with F2; lines intersect at y = 0, y-axis intercept = 0 | 1) F1 = A at or before CLS and ( a) F2 = C, or b) F2 = M before or at CLS) |
| 24 |  | linear; slope and y-intercept increase with F2; lines intersect at y=0, y-axis intercept > 0 (i.e. lines intersect at negative x) | 1) F1 = A after CLS and F2 = A or PU or M, before or at CLS; or 2) F1 = PU before or at CLS and ( a) F2 = A, or b) F2 = PU or M, before or at CLS) |
| 25 |  | linear; slope and y-axis intercept decrease with F2; lines intersect at y = 0, y-axis intercept > 0 | 1) F1 = A after CLS and F2 = C or M, before or at CLS; or 2) F1 = PU before or at CLS and F2 = C or M, before or at CLS |
| 26 |  | linear; slope increases and y-axis intercept fixed with F2; lines intersect at y > 0, y-axis intercept > 0 | 1) F1 = A after CLS and F2 = A after CLS and after F1 |
| 27 |  | linear; slope and y-intercept increases with F2; lines intersect at y > 0, y-axis intercept > 0 | 1) F1 = A after CLS and F2 = A after CLS and before F1 |
| 28 |  | linear; slope decreases and y-axis intercept fixed with F2; lines intersect at y > 0, y-axis intercept > 0 | 1) F1 = A after CLS and F2 = C after CLS at or after F1 |
| 29 |  | linear; slope and y-intercept decrease with F2; lines intersect at y > 0, y-axis intercept > 0 | 1) F1 = A after CLS and F2 = C after CLS and before F1 |
| 30 |  | nonlinear; increasing curve; curve position increases with F2 | 1) F1 = PN or PM, before or at CLS and ( a) F2 = A, or b) F2 = PU or PM, before or at CLS) |
| 31 |  | nonlinear; increasing curve; curve position decreases with F2 | 1) F1 = PN or PM, before or at CLS and ( a) F2 = M or N, before or at CLS, or b) F2 = C) |
| 32 |  | nonlinear; decreasing curve that approaches zero for large F1; curve position increases with F2, | 1) F1 = C before or at CLS and ( a) F2 = A, or b) F2= PU or PM, before or at CLS) |
| 33 |  | nonlinear; decreasing curve that approaches positive value for large F1; curve position increases with F2 | 1) F1 = PM or PN, before or at CLS and ( a) F2 = A, or b) F2= PU or PM, before or at CLS); 2) F1 = C after CLS and ( a) F2 = A, or b) F2= PU or PM, before or at CLS) |
| 34 |  | nonlinear; decreasing curve that approaches zero for large F1; curve position decreases with F2 | 1) F1 = C before or at CLS and ( a) F2 = C, or b) F2 = M or N, before or at CLS) |
| 35 |  | nonlinear; decreasing curve that approaches positive value for large F1; curve position decreases with F2 | 1) F1 = PM or PN, before or at CLS and ( a) F2 = C, or b) F2 = M or N, before or at CLS); or 2) F1 = C after CLS and ( a) F2 = C, or b) F2 = M or N, before or at CLS) |
|  |  |  |  |
| 36 | EC_50_/A_max_ vs. F1 for different values of F2 | linear;  slope increasing with F2 | 1) F1 = C before or at CLS and ( a) F2 = C, or b) F2 = M or N, before or at CLS) |
| 37 |  | linear;  slope decreasing with F2 | 1) F1 = C before or at CLS and ( a) F2 = A, or b) PU or PM, before or at CLS) |
| 38 | EC_50_ vs. F1 | linear, no change with F2 | 1) F1 = C and F2 = at CLS |

Notation used is A = activator, C = competitive inhibitor, U = uncompetitive inhibitor, N = noncompetitive inhibitor, M = mixed inhibitor, L = linear inhibitor, P = partial inhibitor. In Table 1B, plot properties are listed by type of curve (e.g. linear or nonlinear), how the curve changes as F2 changes, and the characteristics of the intersection points and intercepts of the family of curves. Multiple letter activities supersede single letter ones so that, for example, LN is also L. When multiple letter activities, followed by a comma, are listed before a description of the site of activity that means that the site description applies to all of the activities. Of all of the possible scenarios, the most likely are completely described below, which represents over 74% of all combinations.

Notes:

1) 1/EC_50_ plots with no change in position of curves with F2 can be interpreted via Table 1A with F2 = A at CLS

2) any apparently linear plot with a negative slope is nonlinear by mathematical necessity

3) all references to y ≥ 0 refer to the position on the graphs where the total factor (endogenous plus transfected) on the x-axis equals zero

4) y-intercept of A_max_/EC_50_ cannot be < 0 while y-intercept of 1/EC_50_ is always ≥ 0

5) curve position increases or decreases means the sum of the y coordinates of each curve increases or decreases

6) PU is like an activator acting after a local CLS and is indistinguishable from an activator acting after the last and global CLS
