# Supplementary material for: Deducing the Temporal Order of Cofactor Function in Ligand-Regulated Gene Transcription: Theory and Experimental Verification
Source: PLoS One. 2012 Jan 17;7(1):e30225. doi: 10.1371/journal.pone.0030225 (PMC3260260; doi:10.1371/journal.pone.0030225)
Supplement: Text S1 — Derivation of the graphical method for analyzing the competitive action of factors and Correction for non-linear protein expression from transfected plasmids. (DOCX) [file pone.0030225.s006.docx]

**Supporting Information for**

Deducing the temporal order of cofactor function in ligand-regulated gene transcription: theory and experimental verification

Edward J. Dougherty^1^, Chunhua Guo^1^, S. Stoney Simons, Jr.^1^*, and Carson C. Chow^2^*

From the ^2^Laboratory of Biological Modeling, NIDDK, and ^1^Steroid Hormones Section, NIDDK/CEB, National Institutes of Health, Bethesda, MD

**Table of Contents**

- Derivation of the graphical method for analyzing the competitive actions of factors

- Correction for non-linear protein expression from transfected plasmids

*Derivation of the graphical method for analyzing the competitive actions of factors*: Our graphical method stems from our theory of steroid-regulated gene induction, which calculates the dose-response curve for a sequence of *n* reactions [1]. Each reaction step has the form

where *Y*_i_ is the reaction product of step *i*, *X*_i_ is an activating cofactor or activator, *I*_i_ is an inhibiting cofactor or inhibitor. We denote *competitive* inhibition, *uncompetitive* inhibition, *noncompetitive* inhibition, linear inhibition, and *partial* inhibition. Partial inhibition can be activating if it diverts the output to a higher yielding pathway. The dose response curve is obtained by solving the steady state equations for mass conservation and mass action. In general, these equations cannot be solved to obtain a closed form expression for the dose response function of the protein product as a function of the steroid concentration. However, experimentally, it is well established that the dose response curve is closely modeled by a first order Hill function (i.e. Hill coefficient of one). We showed that the dose-response curve for a sequence of *n* such reactions is a first-order Hill function if reaction products have low concentrations or are short-lived [1]. As shown in Ong et al. (2010), this assumption renders the steady state equations for mass conservation and mass action into a solvable form.

The first-order Hill constraint also makes the calculation of the dose-response curve tractable and a formula can be written down explicitly in terms of all the parameters of all reaction steps. The constraint also allows intermediate and unknown reaction steps to “telescope” into effective reactions with effective parameters. Hence, for experiments involving a small number of added cofactors, a model for the dose-response curve can be generated that includes just the action of those cofactors. Within a sequence of reactions, there may be a distinguishing step we termed the Concentration Limiting Step (CLS). The CLS is defined as a step where sets of reactions immediately following it have the property that the amount of cofactor bound to a reaction product is negligible compared to the free concentration. The action of a cofactor is different if it acts before, at or after the CLS and a different parametric model exists for each of these cases.

The parametric models have the abstract form

$[P]=\frac{\Gamma V_{1}^{cls}[S]}{1+W_{1}^{cls}[S]}$ (S1)

where [S] is the steroid concentration, $\Gamma=\sum_{k=cls}^{n} a_{k-cls}V_{cls+1}^{k}$, $V_{b}^{m}=\prod_{i=b}^{m} v_{i}$, and $W_{b}^{m}=\sum_{i=b}^{m} w_{i}V_{b}^{i-1}$. The parameters ν_i_ and ω_i_, which depend on the parameters of the reactions and differ depending on where they act with respect to the CLS, are given in Table S2, where *X^T^* is the total concentration of an added factor and the other parameters come from the reaction given above. The details of the calculation are given in Ong et al. (2010).

Table S2. Parameter expressions

| **Position** | **Activator** | **Activator with Inhibitor** |
| --- | --- | --- |
| Before CLS $i<cls$ | $v_{i}=q_{i}X_{i}^{T}$ $w_{i}=q_{i}\varepsilon_{i}$ | $v_{i}=\frac{q_{i}X_{i}^{T}(1+\alpha_{i}\beta_{i}q'_{i}[I_{i}])}{1+\gamma_{i}q'_{i}[I_{i}]}$ $w_{i}=\frac{q_{i}(\varepsilon_{i}+\alpha_{i}q'_{i}[I_{i}])}{1+\gamma_{i}q'_{i}[I_{i}]}$ |
| At CLS $i=cls$ | *v_i_* same as before CLS $w_{i}=q_{i}\sum_{k=i}^{n} \varepsilon_{k}\prod_{j=i+1}^{k} v_{j}$ | *v_i_* same as before CLS $w_{i}=\frac{q_{i}\left(\sum_{k=i}^{n} \varepsilon_{k}\prod_{j=i+1}^{k} v_{j}+\alpha_{cls}q'_{cls}[I_{cls}]\right)}{1+\gamma_{cls}q'_{cls}[I_{cls}]}$ |
| After CLS $i>cls$ | *v_i_* same as before CLS $w_{i}=0$ | $v_{i}=\frac{q_{i}X_{i}^{T}}{1+\gamma_{i}q'_{i}[I_{i}]}$ $w_{i}=0$ |

All combinations of cofactors are accounted for in the general model (S1). To render the general model into a specific parametric form for a given case, we re-express (S1) such that only the concentrations of the desired factors are explicitly visible. All other reactions “telescope” into an effective reaction with a set of effective parameters. For example, if we are interested in an activator acting somewhere before the CLS then we rewrite $\Gamma V_{1}^{cls}$ as $BX^{T}$, where *B* is a positive parameter that depends on a complicated combination of reaction parameters. The crucial point is that the mechanism of the cofactor can be deduced without knowing the precise value of *B*. Likewise, we can write $W_{1}^{cls}=C+DX^{T}$, for positive constants *C* and *D*. Hence, the dose-response curve for a single activator acting before the CLS has the form

$$[P]=\frac{BX^{T}[S]}{1+(C+DX^{T})[S]}$$

which implies that $A_{\max}=BX^{T}/(C+DX^{T})$, and $EC_{50}=1/(C+DX^{T})$. Hence, for an activator, the graph of A_max_/EC_50_ vs *X^T^* is linear with a positive slope and zero y-intercept and the graph of 1/EC_50_ vs. *X^T^* is a linear function with positive slope and nonzero y-intercept. This can then be repeated for all positions and all co-factor types. The computations to isolate cofactors are facilitated with the decomposition rule

$$W_{a}^{b}=W_{a}^{c}+V_{a}^{c}W_{c+1}^{b}$$

The identical process can be repeated for two factors. In fact all the conclusions for a single factor can be obtained from the parametric models for two cofactors. Table S3 gives the full parametric expressions for two cofactors in all the possible configurations.

Table S3. Decomposition rules

| **Positions** | **Decompositions** | ***B* parameters** |
| --- | --- | --- |
| *g<h<cls*  both before CLS | $V_{1}^{cls}=V_{1}^{g-1}V_{g+1}^{h-1}V_{h+1}^{cls}v_{g}v_{h}$ $\begin{matrix} W_{1}^{cls}=W_{1}^{g-1} \\ \begin{matrix} & \end{matrix}+V_{1}^{g-1}\left(w_{g}+v_{g}\left(W_{g+1}^{h-1}+V_{g+1}^{h-1}\left(w_{h}+v_{h}W_{h+1}^{cls}\right)\right)\right) \end{matrix}$ | $\Gamma V_{1}^{cls}=B_{2}v_{g}v_{h}$ $\begin{matrix} W_{1}^{cls}=B_{3}+B_{4}w_{g}+B_{5}v_{g} \\ \begin{matrix} & \end{matrix}+B_{6}v_{g}w_{h}+B_{7}v_{g}v_{h} \end{matrix}$ |
| *g <h =cls*  before, at CLS | $V_{1}^{cls}=V_{1}^{g-1}V_{g+1}^{cls-1}v_{g}v_{cls}$ $W_{1}^{cls}=W_{1}^{g-1}+V_{1}^{g-1}\left(w_{g}+v_{g}\left(W_{g+1}^{cls-1}+V_{g+1}^{cls-1}w_{cls}\right)\right)$ | $\Gamma V_{1}^{cls}=B_{2}v_{g}v_{h}$ $\begin{matrix} W_{1}^{cls}=B_{3}+B_{4}w_{g}+B_{5}v_{g} \\ \begin{matrix} & & \end{matrix}+B_{6}v_{g}w_{cls} \end{matrix}$ |
| *g <cls< h*  CLS between | $V_{1}^{cls}=V_{1}^{g-1}V_{g+1}^{cls}v_{g}$ $W_{1}^{cls}=W_{1}^{g-1}+V_{1}^{g-1}\left(w_{g}+v_{g}W_{g+1}^{cls}\right)$ $\Gamma=\sum_{k=cls}^{h-1} a_{k-cls}V_{cls+1}^{k}+\left(V_{cls+1}^{h-1}\sum_{k=h}^{n} a_{k-cls}V_{h+1}^{k}\right)v_{h}$ | $\Gamma V_{1}^{cls}=B_{1}v_{g}+B_{2}v_{g}v_{h}$ $W_{1}^{cls}=B_{3}+B_{4}w_{g}+B_{5}v_{g}w_{cls}$ |
| *g=cls<h*  at, after CLS | $V_{1}^{cls}=V_{1}^{cls-1}v_{cls}$ $W_{1}^{cls}=W_{1}^{cls-1}+V_{1}^{cls-1}w_{cls}$ $\Gamma=\sum_{k=cls}^{h-1} a_{k-cls}V_{cls+1}^{k}+\left(V_{cls+1}^{h-1}\sum_{k=h}^{n} a_{k-cls}V_{h+1}^{k}\right)v_{h}$ | $\Gamma V_{1}^{cls}=B_{1}v_{cls}+B_{2}v_{cls}v_{h}$ $W_{1}^{cls}=B_{3}+B_{4}w_{cls}$ |
| *cls<g<h*  both after CLS | $V_{1}^{cls}$ and $W_{1}^{cls}$ are unaffected $\begin{matrix} \Gamma=\sum_{k=cls}^{g-1} a_{k-cls}V_{cls+1}^{k}+\left(V_{cls+1}^{g-1}\sum_{k=g}^{h-1} a_{k-cls}V_{g+1}^{k}\right)v_{g} \\ \begin{matrix} & \end{matrix}+\left(V_{cls+1}^{g-1}V_{g+1}^{h-1}\sum_{k=h}^{n} a_{k-cls}V_{h+1}^{k}\right)v_{g}v_{h} \end{matrix}$ | $\Gamma V_{1}^{cls}=B_{0}+B_{1}v_{g}+B_{2}v_{g}v_{h}$ $W_{1}^{cls}=B_{3}+B_{4}w_{cls}$ |

Expressions for A_max_ and EC_50_ can then be obtained for all cases by inserting the expressions from Table S3 into the general model (equation S1). The results can be summarized in the following five cases:

1. k < l < CLS

$$\frac{A_{\max}}{EC_{50}}=B_{2}v_{k}v_{l}$$

$$\frac{1}{EC_{50}}=B_{3}+B_{4}w_{k}+B_{5}v_{k}+B_{6}v_{k}w_{l}+B_{7}v_{k}v_{l}$$

$$\frac{A_{\max}}{EC_{50}}=B_{2}\frac{q_{l}X_{l}^{T}(1+\alpha_{l}\beta_{l}q'_{l}[I_{l}])}{1+\gamma_{l}q'_{l}[I_{l}]}\frac{q_{k}X_{k}^{T}(1+\alpha_{k}\beta_{k}q'_{k}[I_{k}])}{1+\gamma_{k}q'_{k}[I_{k}]}$$

$$\frac{1}{EC_{50}}=B_{3}+B_{4}\frac{q_{k}(\varepsilon_{k}+\alpha_{k}q'_{k}[I_{k}])}{1+\gamma_{k}q'_{k}[I_{k}]}+\left(B_{5}+B_{6}\frac{q_{l}(\varepsilon_{l}+\alpha_{l}q'_{l}[I_{l}])}{1+\gamma_{l}q'_{l}[I_{l}]}+B_{7}\frac{q_{l}X_{l}^{T}(1+\alpha_{l}\beta_{l}q'_{l}[I_{l}])}{1+\gamma_{l}q'_{l}[I_{l}]}\right)\frac{q_{k}X_{k}^{T}(1+\alpha_{k}\beta_{k}q'_{k}[I_{k}])}{1+\gamma_{k}q'_{k}[I_{k}]}$$

1. k < l = CLS

$$\frac{A_{\max}}{EC_{50}}=B_{2}v_{g}v_{h}$$

$$\frac{1}{EC_{50}}=B_{3}+B_{4}w_{k}+B_{5}v_{k}+B_{6}v_{k}w_{cls}$$

$$\frac{A_{\max}}{EC_{50}}=B_{2}\frac{q_{l}X_{l}^{T}(1+\alpha_{l}\beta_{l}q'_{l}[I_{l}])}{1+\gamma_{l}q'_{l}[I_{l}]}\frac{q_{k}X_{k}^{T}(1+\alpha_{k}\beta_{k}q'_{k}[I_{k}])}{1+\gamma_{k}q'_{k}[I_{k}]}$$

$$\frac{1}{EC_{50}}=B_{3}+B_{4}\frac{q_{k}(\varepsilon_{k}+\alpha_{k}q'_{k}[I_{k}])}{1+\gamma_{k}q'_{k}[I_{k}]}+\left(B_{5}+B_{6}\frac{q_{l}\left(B_{7}+\alpha_{l}q'_{l}[I_{l}]\right)}{1+\gamma_{l}q'_{l}[I_{l}]}\right)\frac{q_{k}X_{k}^{T}(1+\alpha_{k}\beta_{k}q'_{k}[I_{k}])}{1+\gamma_{k}q'_{k}[I_{k}]}$$

1. k< CLS < l

$$\frac{A_{\max}}{EC_{50}}=B_{1}v_{k}+B_{2}v_{k}v_{l}$$

$$\frac{1}{EC_{50}}=B_{3}+B_{4}w_{k}+B_{5}v_{k}w_{cls}$$

$$\frac{A_{\max}}{EC_{50}}=\left(B_{1}+B_{2}\frac{q_{l}X_{l}^{T}}{1+\gamma_{l}q'_{l}[I_{l}]}\right)\frac{q_{k}X_{k}^{T}(1+\alpha_{k}\beta_{k}q'_{k}[I_{k}])}{1+\gamma_{k}q'_{k}[I_{k}]}$$

$$\frac{1}{EC_{50}}=B_{3}+B_{4}\frac{q_{k}(\varepsilon_{k}+\alpha_{k}q'_{k}[I_{k}])}{1+\gamma_{k}q'_{k}[I_{k}]}+\left(B_{5}+B_{6}\frac{q_{l}X_{l}^{T}}{1+\gamma_{l}q'_{l}[I_{l}]}\right)\frac{q_{k}X_{k}^{T}(1+\alpha_{k}\beta_{k}q'_{k}[I_{k}])}{1+\gamma_{k}q'_{k}[I_{k}]}$$

4. k = CLS < l

$$\frac{A_{\max}}{EC_{50}}=B_{1}v_{k}+B_{2}v_{k}v_{l}$$

$$\frac{1}{EC_{50}}=B_{3}+B_{4}w_{k}$$

$$\frac{A_{\max}}{EC_{50}}=\left(B_{1}+B_{2}\frac{q_{l}X_{l}^{T}}{1+\gamma_{l}q'_{l}[I_{l}]}\right)\frac{q_{k}X_{k}^{T}(1+\alpha_{k}\beta_{k}q'_{k}[I_{k}])}{1+\gamma_{k}q'_{k}[I_{k}]}$$

$$\frac{1}{EC_{50}}=B_{3}+B_{4}\frac{q_{k}}{1+\gamma_{k}q'_{k}[I_{k}]}\left(B_{5}+B_{6}\frac{q_{l}X_{l}^{T}}{1+\gamma_{l}q'_{l}[I_{l}]}+\alpha_{k}q'_{k}[I_{k}]\right)$$

1. CLS < k< l

$$\frac{A_{\max}}{EC_{50}}=B_{0}+B_{1}v_{k}+B_{2}v_{k}v_{l}$$

$$\frac{1}{EC_{50}}=B_{3}+B_{4}w_{cls}$$

$$\frac{A_{\max}}{EC_{50}}=B_{0}+\left(B_{1}+B_{2}\frac{q_{l}X_{l}^{T}}{1+\gamma_{l}q'_{l}[I_{l}]}\right)\frac{q_{k}X_{k}^{T}}{1+\gamma_{k}q'_{k}[I_{k}]}$$

$$\frac{1}{EC_{50}}=B_{3}+B_{4}\frac{q_{k}X_{k}^{T}}{1+\gamma_{k}q'_{k}[I_{k}]}+B_{5}\frac{q_{l}X_{l}^{T}}{1+\gamma_{l}q'_{l}[I_{l}]}$$

All of the *B* parameters are positive numbers. From these five cases we can then extract the graphical behavior of A_max_/EC_50_ vs cofactor and 1/EC_50_ vs cofactor for all cofactor types, where $X_{i}^{T}$is the concentration of an activator at step *i*, $[I_{i}]$ is the concentration of the inhibitor at step *i*, and the type of inhibitor is determined by the parameters $\alpha_{i}$, $\beta_{i}$, and $\gamma_{i}$. These conclusions are listed in Tables 1 and 2. Note that decreasing curves are always first order decay plots.

*Correction for non-linear protein expression from transfected plasmids*: Western blots reveal a non-linear relationship between the optical density of scanned protein band and the amount of transfected plasmid at constant levels either of total cellular protein or of an internal standard, such as $\beta$-actin. All of the plots of this study require a linear relationship for the amount of factor given on the x-axis. To determine the linear equivalent of expressed plasmid, the non-linear plot of OD vs. ng of transfected plasmid is first fit to a Michaelis-Menten plot (**Fig. S1A**) of

*A_max_ = m1*plasmid/(m2 + plasmid)*

The functional equivalent of the transfected plasmid that gives a linear OD vs. plasmid plot is then obtained from the formula of

*Plasmid (linear) = m2*plasmid/(m2 + plasmid)*

The amount of plasmid plotted in the various graphs is then this “corrected plasmid” value (**Fig. S1B**).

*Matching graphs to graphical descriptions*: The following algorithms are used to determine which graphical description in Table 1 best describes a given plot.

1) Examination of the underlying equations (see above) indicates that, for all plots, a negative slope can only be fit to a non-linear MIchaelis-Menten decay curve.

2) Whether a plot with a positive slope is linear or non-linear (and curving downward) is based upon the reproducibility of the fit to a straight line, both for all four lines within a given graph and for all such graphs for a given competition experiment. Random fluctuations will occasionally yield data points for one line that may appear to be best described by a downward bending curve. However, if this behavior is not consistently observed, both for the other three plots of the same graph and for the plots of the other experiments under the same conditions, the plot is deemed to be best fit by a straight line.

3) In order to decide whether the nonlinear, decreasing curves for A_max_/EC_50_ go to zero (entries 32 or 34 of Table S1) or to a positive plateau value (entries 33 or 35 of Table 1b), one looks at the reciprocal plots (EC_50_/A_max_ vs. F1) (2). If the reciprocal plots are straight lines, then the curves of the A_max_/EC_50_ graph go to zero at infinite F1. If the reciprocal plots curve downward, but give linear plots when first subtracting a probable asymptote from the value of A_max_/EC_50_ and then plotting the reciprocal this new value (= 1/[(A_max_/EC_50_) – asymptote]), then the A_max_/EC_50_ plot goes to that asymptote at infinite F1

4) A final criterion for classifying the nature of a given plot comes from the analysis of the graphical interpretations of the 4-6 plots utilized for a given competition experiment. The final interpretation must be consistent with the partial interpretation derived from each type of graph. No one graph can uniquely define all of the relationships of the two factors and their kinetic mechanism. Rather the assembly of graphs, and their partial interpretations, are used to construct the final mechanism. In this respect, the final mechanism is that area of common overlap of the possible scenarios of each type of graph. This is pictorially represented in Fig. S4. Each circle represents one type of graph, with sectors of each circle representing the different possible mechanisms associated with the graph. By identifying that scenario of each graph that is consistent with all of the other graphs (indicated by the filled area where all six circles overlap), one usually arrives at a unique mechanistic explanation. If two descriptions of one graph appear to be equally possible but only one gives “overlap” with the other 3-5 plots, then this “overlapping” plot is selected.

References

1. Ong KM, Blackford Jr JA, Kagan BL, Simons Jr SS, Chow CC (2010) A new theoretical framework for gene induction and experimental comparisons. Proc Natl Acad Sci U S A 107: 7107-7112.

2. Chow CC, Ong KM, Dougherty EJ, Simons, Jr. SS (2011) Inferring mechanisms from dose-response curves. Methods in Enzymology 487: 465-483.
